# Alternate Day Fasting Enhances Intestinal Epithelial Function During Aging by Regulating Mitochondrial Metabolism

**DOI:** 10.1111/acel.70052

**Published:** 2025-04-01

**Authors:** Heng Quan, Yao Lu, Yingying Lin, Peng Xue, Yuning Zhang, Yuqi Wang, Weiru Yu, Xiaoya Lin, Wuqi Yang, Cong Lv, Yafei Zhang, Fazheng Ren, Huiyuan Guo

**Affiliations:** ^1^ Key Laboratory of Functional Dairy, Department of Nutrition and Health China Agricultural University Beijing China; ^2^ College of Food Science and Nutritional Engineering China Agricultural University Beijing China

**Keywords:** aging, alternate day fasting, gut, intestinal stem cell, mitochondrial metabolism

## Abstract

With advancing age, the decline in intestinal stem cell (ISC) function can lead to a series of degenerative changes in the intestinal epithelium, a critical factor that increases the risk of intestinal diseases in the elderly. Consequently, there is an urgent imperative to devise effective dietary intervention strategies that target the alterations in senescent ISCs to alleviate senescence‐related intestinal dysfunction. The 28‐month‐old naturally aging mouse model was utilized to discover that the primary factor contributing to the compromised barrier function and digestive absorption of the small intestine was a decrease in both the number and regenerative capacity of ISCs. The underlying mechanism involves the degeneration of mitochondrial function in ISCs, resulting in insufficient energy supply and decreased metabolic capacity. Additionally, our findings indicate that fasting‐refeeding can influence the mitochondrial metabolism of ISCs, and that alternate day fasting (ADF) can facilitate the restoration of both the quantity and regenerative capabilities of ISCs, thereby exhibiting a notable antiaging effect on the small intestine. In conclusion, this study provides new insights into the potential beneficial role of ADF in ameliorating intestinal aging, thereby establishing a foundation for future investigations into dietary interventions aimed at addressing age‐related intestinal dysfunction.

## Graphical abstract

Alternate day fasting alleviates aging‐induced intestinal epithelial barrier damage by regulating mitochondrial metabolism in small intestinal stem cells.

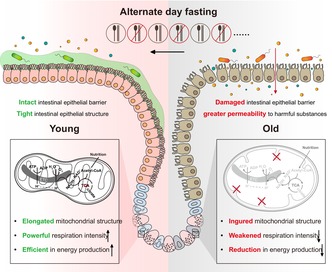

**Abbreviations**
2‐DG: 2‐deoxyglucose
ADF: alternate day fasting
AL: ad libitum
ATP: Adenosine Triphosphate
DAO: diamine oxidase
DLA: D‐lactic acid
ECAR: extracellular acidification rate
EDTA: ethylenediaminetetraacetic acid
FAO: fatty acid oxidation
FITC: fluorescein iso‐thiocyanate
GSEA: Gene Set Enrichment Analysis
H&E: hematoxylin and eosin
IEC: intestinal epithelial cell
ISC: intestinal stem cell
LC–MS: liquid chromatography‐mass spectrometry
mtDNA: mitochondrial DNA
mTORC1: mammalian target of rapamycin complex 1
OAT: ornithine aminotransferase
OCR: oxygen consumption rate
OXPHOS: oxidative phosphorylation
PBS: phosphate buffered saline
SIRT1: silent information regulator 1

## 1 Introduction

The small intestine, a vital component of the digestive system, is responsible for critical functions related to digestion and nutrient absorption, in addition to fulfilling significant barrier and secretory roles. As individuals age, the small intestine experiences a range of degenerative changes that lead to the development of numerous gastrointestinal disorders commonly observed in the elderly population. A notable consequence of aging is the alteration of the epithelial structure within the small intestine, which results in a reduced capacity of the intestinal villi to absorb nutrients. This deterioration in absorptive capability may consequently lead to malnutrition in the elderly (Woudstra and Thomson 2002). Moreover, the aging process often compromises the selective permeability of the small intestinal epithelial barrier, permitting the unregulated entry of contents from the intestinal lumen into the systemic circulation (Kawamoto and Hara 2024). This occurrence can instigate chronic inflammation, potentially exacerbating the aging process.

Intestinal stem cells (ISCs), located in proximity to Paneth cells at the base of the small intestinal crypts (Gehart and Clevers 2019), serve as the primary facilitators of intestinal epithelial regeneration. They are essential for maintaining the population of intestinal cells and for upholding the structural and functional integrity of the intestinal barrier. It is increasingly acknowledged that the functionality of ISC declines with age. A significant factor contributing to the aging of ISCs is the reduction of classical Wnt signaling pathways. Research has demonstrated that the supplementation of Wnt3a in organoid culture media can enhance Wnt signal transduction in ISCs, thereby markedly improving their organoid formation capacity (Nalapareddy et al. 2017). Additionally, research has demonstrated that the activity of the silent information regulator 1/mammalian target of rapamycin complex 1 (SIRT1/mTORC1) pathway declines with age; however, reactivation of this pathway through the administration of the NAD^+^ precursor nicotinamide riboside may enhance the regenerative capacity of the intestine in older individuals (Igarashi et al. 2019). There are also specific inhibitors available that could potentially alleviate the age‐related decline in ISC functionality. Furthermore, there is an ongoing discussion regarding the irregularities associated with the fundamental roles of ISCs, which include self‐renewal, differentiation, and metabolic processes, in naturally aging murine. It is crucial to investigate the mechanisms that underlie the decline in small intestine function related to aging, particularly through the regulation of ISC activity. This research is vital for the improvement of digestive and absorptive functions, with the ultimate goal of enhancing the quality of life for the elderly.

In recent years, restrictive diets have emerged as the primary nutritional intervention strategy with the potential to effectively extend lifespan (Nakada et al. 2011). Alternate day fasting (ADF) is a dietary pattern characterized by periodic alternation between normal caloric intake and caloric restriction. This approach mitigates the adverse effects associated with caloric restriction and presents a more advantageous strategy for the organism by integrating the beneficial aspects of fasting physiology with the promotion of tissue repair and regeneration during the feeding phase. Current research suggests that a 24‐h fasting period can enhance fatty acid oxidation, thereby improving the function of ISCs in both young and aged mice (Mihaylova et al. 2018). Additionally, short‐term fasting followed by refeeding has been shown to enhance ISC viability by promoting polyamine metabolism (Imada et al. 2024). Furthermore, resuming a normal diet after short‐term fasting appears to be beneficial for inflammation levels, stem cell proliferation, and tissue maintenance within the small intestine (de Cabo and Mattson 2019). These studies consistently demonstrate that ADF positively influences the maintenance of intestinal epithelial cells. Nevertheless, the impact of ADF on the small intestinal epithelium in older adults is not yet well understood. Furthermore, the mechanisms that connect the fate of ISC to fluctuations in dietary intake necessitate additional research.

In this study, we employed naturally aged mice alongside a rapidly aging mouse model to examine the changes occurring in the small intestinal epithelium and the functionality of ISCs throughout the aging process. Our results demonstrate that an ADF dietary regimen can alleviate the aging of ISCs. Additionally, we clarify the mechanistic connections between dietary patterns and ISC functionality. These findings suggest that future dietary intervention strategies for older adults could be optimized to counteract the age‐related deterioration of ISC function, thereby enhancing normal bowel function within the aging demographic.

## 2 Materials and Methods

### 2.1 Ethics

All mouse experimental procedures and protocols were approved by the Beijing Laboratory Animal Management and were conducted in strict accordance with the guidelines set forth by the Institutional Animal Care and Use Committee of China Agricultural University (approval number: AW91904202‐5‐1).

### 2.2 Mice

In this study, male C57BL/6J mice were maintained in specific pathogen‐free facilities under a controlled light–dark cycle of 12:12 h (08:00–20:00), with a humidity level of 45% ± 5%, a temperature range of 22°C–24°C, and ad libitum access to food and water. Lgr5‐EGFP‐IRES‐CreERT2 mice were acquired from Shanghai Model Biology. The experimental cohorts comprised young mice (2–3 months old) and naturally aged mice (24–28 months old), representing distinct physiological stages of intestinal aging.

### 2.3 Rapid Aging Mouse Model

D‐galactose‐induced mice were utilized to establish a rapid aging model. In this experiment, 8‐week‐old C57BL/6J mice received continuous intraperitoneal injections of 500 mg/kg D‐galactose (Sigma‐Aldrich G5388) over 2 months.

### 2.4 Alternate Day Fasting Model

Eight‐week‐old mice were randomly assigned to two groups: AL and ADF. ADF was conducted by withholding food for 24 h (12:00 PM to 12:00 PM the following day), with ad libitum water access maintained throughout the fasting period, thereby establishing a 24‐h cycle of fasting and feeding. Mice in the control group received fresh food at the same time each day. The body weight of each mouse, as well as the total food weight for each cage, was recorded daily to calculate the average daily food intake per mouse. The final phase of the study commenced at 9:00 AM and concluded at 11:00 AM, following a period of overnight feeding or fasting.

### 2.5 Crypt Isolation and Organoid Culturing

Organoids are generated from crypts which are extracted from the small intestine. In brief, the murine small intestine was incised along the longitudinal axis, and the villi were scraped off before being placed into ice‐cold PBS. The tissue was washed five times until the supernatant was nearly clear, after which the intestine was shaken in ice‐cold PBS containing 5 mM EDTA for 15 min. Following the removal of the digestive fluid, the intestine was vigorously shaken, and the crypts were collected, passed through a 70 μm cell strainer, and centrifuged. The crypts were then resuspended with Dulbecco's Modified Eagle Medium/Nutrient Mixture F‐12 (DMEM/F12, Gibco), centrifuged again, and seeded into 48‐well plates. Each well contained 30 μL droplets, prepared by mixing GM medium (StemCell) and Matrigel (Corning) in a ratio of 4:6. After 5 days of crypt culture at 37°C and 5% CO_2_, the primary generation of organoids was formed. For the passaging of organoids, the droplets were disrupted using ice‐cold PBS. We repeated pipetting until the organoid buds were sufficiently disaggregated. Following this, the mixture should be centrifuged, after which the organoids can be resuspended and seeded into a new plate to ensure optimal growth conditions.

### 2.6 Flow Cytometry

Intestinal crypts isolated from Lgr5‐GFP mice were incubated in a digestion solution containing 1.5 U/mL Dispase II (Roche) and DNase I (TIANGEN, RT411) for 4 min to generate a single‐cell suspension. The dissociated cells were filtered through a 40 μm cell strainer and centrifuged at 500 g for 5 min before being resuspended in 1 mL of PBS containing Hoechst 33342 and incubated on ice for 10 min. A flow cytometer (BD FACSAria SORP) was utilized to detect Lgr5‐positive signals, and the corresponding Lgr5‐positive cells were subsequently collected. Anti‐apoptotic inhibitors were added to the medium. Following centrifugation at 500 g, the resulting pellet was resuspended and seeded into a new plate at a density of 5000 cells per well.

### 2.7 Agilent Seahorse XF

The evaluation of OCR and ECAR in ISCs was conducted using a Seahorse Analyzer. Mitochondrial OCR was measured with the Seahorse XFe24 extracellular flux analyzer, following the protocol established by Agilent Technologies. Primary crypts isolated from the small intestine were seeded into Seahorse XF24 cell culture plates at a density of 30 cells per well. Simultaneously, the sensor cartridge was hydrated overnight in a CO_2_‐free calibration buffer maintained at 37°C. Prior to the experiment, the cells were incubated for 1 h at 37°C in XF‐Base medium supplemented with L‐glutamine in a CO_2_‐free environment. Final concentrations of 1 μM oligomycin, 1 μM FCCP, and 0.5 μM rotenone combined with 0.5 μM antimycin were incorporated into the mitochondrial stress test medium. In the cartridge, 75 μL of the mitochondrial stress test medium mixture was dispensed into each well of ports A, B, and C, respectively. The procedural setup was as Table 1.

**TABLE 1:** Settings mitochondrial stress test.

Settings			Intestine‐derived organoids
Basal	3 cycles	Mix	4 min
		Wait	0 min
		Measure	2 min
Oligomycin	1 cycle	Mix	5 min
		Wait	10 min
		Measure	2 min
	2 cycles	Mix	4 min
		Wait	0 min
		Measure	2 min
FCCP	3 cycles	Mix	4 min
		Wait	0 min
		Measure	2 min
Retenone+Antimycin A	3 cycles	Mix	4 min
		Wait	0 min
		Measure	2 min

In the glycolytic stress test, the methodology employed was consistent with that utilized in the mitochondrial OCR test, with the sole modification being the incorporation of glutamine into the Seahorse XF DMEM as the culture medium. The experimental setup involved the addition of a pharmacological agent in the cartridge, achieving final concentrations of 10 mM glucose, 5 μM oligomycin, and 100 mM 2‐DG over three cycles. Each drug was subjected as Table 2.

**TABLE 2:** Settings glycolysis stress test.

Settings			Intestine‐derived organoids
Basal	3 cycles	Mix	4 min
		Wait	0 min
		Measure	2 min
Glucose	3 cycles	Mix	4 min
		Wait	1 min
		Measure	2 min
Oligomycin	1 cycle	Mix	5 min
		Wait	10 min
		Measure	2 min
	2 cycles	Mix	4 min
		Wait	0 min
		Measure	2 min
2‐DG	3 cycles	Mix	4 min
		Wait	0 min
		Measure	2 min

Upon completion of the procedure, the results were analyzed using Seahorse XF24 Wave software and normalized to total protein concentration.

### 2.8 ^13^C‐Palmitate LC–MS

Isolated intestinal crypts were placed in 500 μL of RPMI medium supplemented with 100 μM 13C‐palmitate per well and incubated at 37°C, 5% CO_2_ for 1 h. Following incubation, the samples were washed and centrifuged with PBS, after which they were collected in a 1.5 mL centrifuge tube. Ice‐cold LC–MS grade methanol, acetonitrile, and water in a ratio of 1:1:2 were added to the samples. The mixture was vortexed for 10 min and subsequently centrifuged at 4°C for 10 min. The resulting pellet was dried using a vacuum desiccator and then resuspended in 100 μL of LC–MS grade water. Pipette 2 μL of the solution onto a ZIC‐pHILIC column (2.1 × 150 mm, particle size 5 μm; EMD Millipore). Buffer A consisted of 20 mM NH_4_HCO_3_ with 0.1% NH_3_‐H_2_O, while Buffer B was composed of acetonitrile. The chromatographic gradient was established as follows: from 0 to 20 min, a linear gradient was applied from 80% Buffer B to 20% Buffer B; from 20 to 20.5 min, a linear gradient was applied from 20% Buffer B back to 80% Buffer B; and from 20.5–30 min, the system was held at 80% Buffer B. Multiple reaction monitoring was conducted using a QExactive Orbitrap mass spectrometer, with spray voltages set at 4500 V (negative mode) and 5500 V (positive mode), a temperature maintained at 500°C, and ion source gases 1 and 2 set at 55 psi.

### 2.9 ATP Measurement

In accordance with the protocol provided by the manufacturer of the ATP assay kit (Beyotime, S0027), the lysate was incorporated into the small intestinal tissue and homogenized thoroughly using a glass homogenizer. Subsequently, the mixture was centrifuged at 4°C at 12,000 g for 5 min, and the supernatant was collected for analysis. The ATP standard solution was diluted to create appropriate concentration gradients of 0.01, 0.03, 0.1, 0.3, 1, 3, and 10 μM. An aliquot of 100 μL of the ATP assay solution was added to the assay well and allowed to incubate at room temperature for 3–5 min to ensure the complete consumption of background ATP. Following this, 20 μL of either the sample or standard was added to the well, mixed rapidly, and the luminescence was measured using a luminometer to determine the ATP concentration in the sample based on the standard curve. Finally, the protein concentration of the sample was assessed, and the ATP concentration was expressed in nmol/mg of protein.

### 2.10 Histology, Immunohistochemistry and Immunofluorescence

Fresh small intestinal tissue was subjected to fixation with formalin, followed by dehydration through a gradient of ethanol and embedding in paraffin. Subsequently, the tissue was sectioned into 5 μm slices. The sections were deparaffinized using xylene I and xylene II for 15 min, and then rehydrated in a series of ethanol solutions: 100% ethanol I, 100% ethanol II, 95% ethanol, 80% ethanol, and 70% ethanol, each for a duration of 5 min. Following this rehydration process, the subsequent staining procedures commenced.

For hematoxylin and eosin (H&E) staining, paraffin‐embedded tissue is sectioned to a thickness of 5 μm. Following deparaffinization in xylene and a gradient of ethanol, the sections are stained with hematoxylin for 30 s and subsequently with eosin for 10 s. The sections are then dehydrated and mounted in neutral resin for observation.

For immunohistochemical staining, tissue sections are deparaffinized using xylene, followed by treatment with serial dilutions of ethanol. Antigen retrieval is achieved by heating the slides in a 0.01 M citrate buffer (pH 6.0) for 30 min in a microwave oven. After allowing the slides to cool to room temperature, a 3% hydrogen peroxide solution is applied to the sections, which are then incubated with a blocking solution for 1 h to inhibit endogenous peroxidase activity. The sections are subsequently incubated overnight at 4°C with the primary antibody. Following this incubation, the sections are immunostained utilizing the peroxidase method, employing diaminobenzidine as the enzyme substrate and hematoxylin as the counterstain. Finally, the sections are dehydrated and mounted in neutral resin for subsequent observation.

For immunofluorescence staining, the sections are prepared according to previously established protocols. Following the incubation with the primary antibody, the sections are subsequently incubated with a secondary antibody (Invitrogen) and counterstained with Hoechst 33342 (Beyotime, C1028) for a duration of 10 min. Finally, the sections are sealed using an antifluorescent quencher. The primary antibodies used in this study include Abcam, ab185230, 1:2000; Abcam, ab15580, 1:1000; Abcam, ab283265, 1:5000; CST, 39141 T, 1:400; Servicebio, GB11344, 1:1000.

### 2.11 DNA Copy Number Measurement

Total DNA was extracted from small intestinal crypts utilizing the TIANamp Micro DNA Kit (TIANGEN) in accordance with the manufacturer's instructions. The mitochondrial DNA (mtDNA) fragment was obtained through PCR amplification, followed by a relative quantitative assessment of the mtDNA copy number, using nuclear DNA (nDNA) as a reference. The primer sequences employed for the mtDNA are as follows:

Nd2‐Forward, 5′‐CACGATCAACTGAAGCAGCAA‐3′.

Nd2‐Reverse, 5′‐ACGATGGCCAGGAGGATAATT‐3′.

Atp6‐Forward, 5′‐AATTACAGGCTTCCGACACAAAC‐3′.

Atp6‐Reverse, 5′‐TGGAATTAGTGAAATTGGAGTTCCT‐3′.

Cytb‐Forward, 5′‐GCCACCTTGACCCGATTCT‐3′.

Cytb‐Reverse, 5′‐TTCCTAGGGCCGCGATAAT‐3′.

Dl‐Forward, 5′‐AATCTACCATCCTCCGTGAAACC‐3′.

Dl‐Reverse, 5′‐GCCCGGAGCGAGAAGAG‐3′.

### 2.12 Fluorescein Iso‐Thiocyanate (FITC) Measurement

Remove approximately 7 cm of small intestine tissue from the mouse without rinsing. Initially, ligate one end of the segment, then inject 200 μL of 0.001 mol/L FITC solution into the other end before completely ligating it. Measure the length between the two knots. Place the ligated intestinal segment in KHBB buffer and incubate it in a 37°C water bath for 20 min. During this process, ensure that the knot remains secure. Finally, measure the fluorescence intensity using a microplate reader.

### 2.13 Transmission Electron Microscopy

Fresh intestinal tissue was subjected to fixation in 2.5% glutaraldehyde (Solarbio) at a temperature of 4°C for a duration of 1 h. Following fixation, the tissue underwent three washes with 0.1 M phosphate buffer, was subsequently fixed in 1% OsO_4_ for an additional hour, and was dehydrated through a sequential increase in acetone concentration (50%, 70%, 80%, 90%, 95% for 15 min each; 100% for two intervals of 20 min). The sample was then embedded and polymerized at 37°C for 12 h, at 45°C for 12 h, and at 60°C for 48 h. Ultrathin sections, measuring 70–100 nm, were prepared and stained with 2% uranyl acetate followed by lead citrate. Imaging was conducted using a JEM‐1200EX electron microscope.

### 2.14 Lactase Activity

Lactase activity was assessed in accordance with the kit protocol. The weight of the small intestine tissue was precisely recorded, and a 1:9 ratio of tissue volume (g) to 0.9% saline was established. The resulting mixture was mechanically homogenized in an ice water bath at 2500 rpm for 10 min, followed by centrifugation for 10 min. The supernatant was collected and combined with the substrate, mixed thoroughly, and incubated at 37°C for 20 min. A terminating agent was subsequently added, and the mixture was mixed well before undergoing centrifugation at 4000 rpm for 10 min. The supernatant was then collected and subjected to colorimetric analysis using a Microplate Reader. The treatment was normalized based on the protein concentration.

### 2.15 Elisa

The determination of serum levels of DAO and D‐lactic acid was conducted in accordance with the specified kit protocol. Collect blood from the mouse and allow it to coagulate naturally at room temperature for 30 min. Centrifuge the sample at 2500 rpm for 15 min and collect the supernatant. Set up standard and sample wells, adding 50 μL of standards at varying concentrations to each standard well. Add the sample and enzyme reagent to the appropriate wells, excluding the blank well. Seal the plate with a sealing film and incubate at 37°C for 60 min. Discard the liquid, shake the plate to dry, and wash it five times. Add the developer and incubate at 37°C for 15 min in the dark. Finally, add the stop solution, zero the blank wells, and measure the absorbance (OD value) of each well sequentially at 450 nm.

### 2.16 JC‐1 Staining

After the digestion of mouse small intestinal epithelial cells into single‐cell suspensions, the cells were centrifuged at 500 g for 5 min, resuspended in 1 mL of PBS, and treated with 20 μL of JC‐1 dye at a cell density of 5 × 10^5^ cells/mL. The cells were incubated at 37°C in a 5% CO_2_ environment for 15–20 min, followed by a second centrifugation at 500 g for 5 min. The cells were then resuspended in 1 mL of PBS and analyzed using a detection machine.

### 2.17 Statistical Analysis

All experiments were conducted independently three times. For organoid assays and in vivo studies, samples from at least three different mice were utilized in each experimental group. Results are presented as mean ± SD. Data analysis was performed using SPSS (IBM), Excel (Microsoft), and GraphPad Prism version 10. ImageJ was employed for image analysis, while FlowJo 10.4 was used for flow cytometry analysis. The significance of the differences between the two groups was analyzed using Student's t‐test. Significant differences were established at a level of *p* < 0.05, with *p < 0.05, **p < 0.01, and ***p < 0.001 indicating varying degrees of significance. Nonsignificant results were denoted as ns. It is important to note that the animal experiments were not conducted in a blinded manner.

## 3 Results

### 3.1 The Functions of the Small Intestine Are Compromised in Aged Mice

**FIGURE 1:**
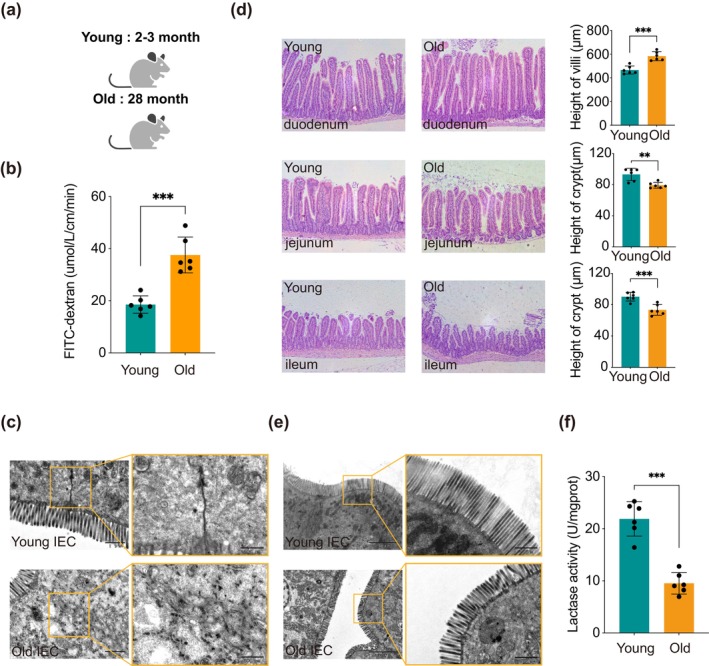
The barrier and digestive functions of the small intestine in aged mice are impaired, and the intestinal epithelial structure is altered. (a) Schematic diagram illustrating the ages of young and old mice. (b) The ability of young and old mice to allow selective permeation of the small intestine was evaluated using the fluorescent dextran probe FITC‐Dextran. (c) Representative images of tight junction protein structures in the small intestines of young and old mice. Scale bars: 1 μm and 500 nm. (d) Representative H&E‐stained sections of the duodenum, jejunum, and ileum of young and old mice, along with statistical analyses of villus height and crypt depth in the three intestinal segments. (e) Representative images of the microvilli structure in the small intestinal epithelium of young and old mice. Scale bars: 5 μm and 1 μm. (f) Lactase activity in young and old mice. Each experiment was performed in triplicate, and the data are presented as mean ± SD. Statistical analysis was conducted using a Student's *t*‐test, with significance levels indicated as *p < 0.05, **p < 0.01, and ***p < 0.001. The age of the young mice was 2–3 months, while the aged mice were 28 months old.

The functions of the small intestine in 28‐month‐old naturally aged mice gradually became disordered (Figure 1a). It was observed that the small intestine could not maintain normal selective permeability, resulting in a significant increase in the leakage rate of the FITC solution (Figure 1b). Additionally, we noted that the tight junction structures between the epithelial cells of the small intestine in aged mice appeared disorganized and indistinct, with instances of tight junction structure breakage (Figure 1c). This indicates that the barrier of the intestinal epithelium in aged mice is compromised. Furthermore, by examining the alterations in villi and crypts across various regions of the small intestine using H&E staining, we observed that the villi in the duodenum of aged mice were elongated, while there were minimal changes in the jejunum and ileum. Additionally, the crypt heights in both the duodenum and ileum were significantly reduced. Although the crypt height in the jejunum did not exhibit a statistically significant difference, it displayed a downward trend (Figure 1d). In addition to observing the overall structural changes of the intestinal epithelium macroscopically, we employed transmission electron microscopy to examine the morphology and arrangement of microvilli in the small intestine at a more microscopic level. Our findings revealed that, compared to the microvilli in the young small intestine, those in the aged small intestine were sparsely arranged, significantly atrophied, and exhibited a tendency to detach (Figure 1e). This observation indicates that aging leads to a marked decline in the absorption capacity of intestinal absorptive cells. Furthermore, the activity of lactase, the primary digestive enzyme in the small intestine, was significantly reduced with aging (Figure 1f). Consequently, the experimental data further demonstrate that the digestive and absorptive functions of the small intestine are impaired as a result of aging.

### 3.2 The Quantity and Functionality of ISC Are Reduced in Aged Mice

**FIGURE 2:**
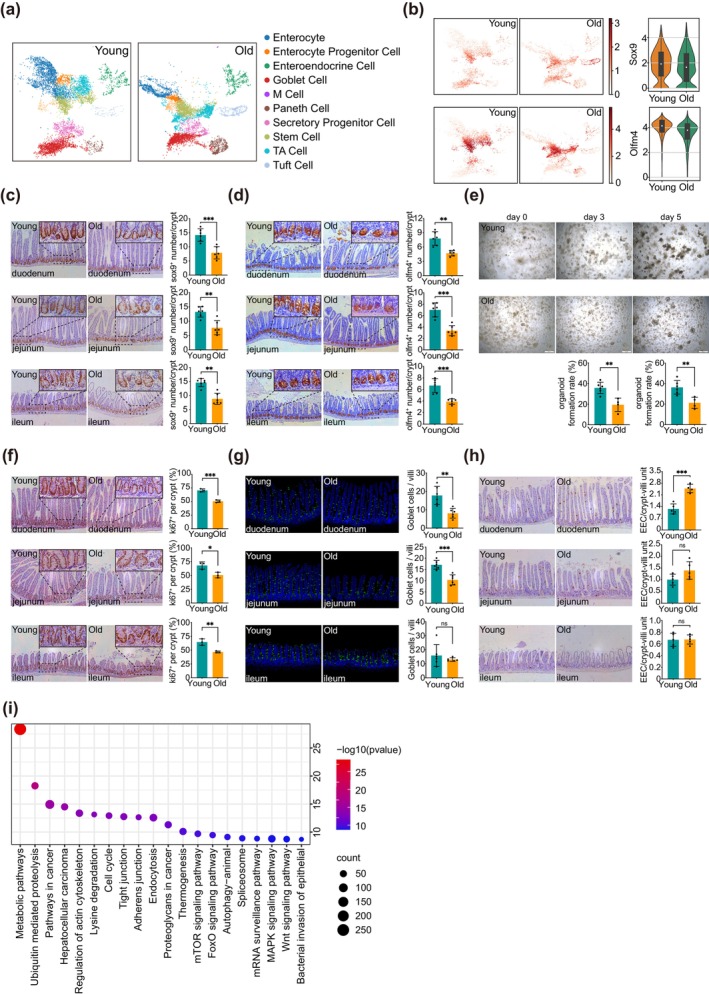
The number of ISCs in aged mice decreases, leading to disruptions in their core functions, including regeneration, proliferation, and differentiation. (a) A UMAP plot illustrates the analysis of single‐cell sequencing data derived from the small intestinal epithelium of both young (2‐month‐old) and aged (28‐month‐old) mice, with *n* = 1 for each group. (b) Results of the single‐cell sequencing analysis of young and old mice: Expression levels of the marker genes Sox9 and Olfm4 in small intestinal stem cells. (c) Representative immunohistochemical staining sections of the duodenum, jejunum, and ileum from young and old mice. The number of Sox9^+^ cells in each crypt of the three intestinal segments was quantified. (d) Representative immunohistochemical staining sections of the duodenum, jejunum, and ileum from young and old mice. The number of Olfm4^+^ cells in each crypt of the three intestinal segments was quantified. (e) Representative images and statistical results of organoids derived from the small intestine of both young and old mice. Scale bar = 200 μm. (f) Representative immunohistochemically stained sections of the duodenum, jejunum, and ileum from young and old mice, including a count of Ki67^+^ cells in each crypt across the three intestinal segments. (g) Representative immunofluorescence‐stained sections of the duodenum, jejunum, and ileum from young and old mice, quantifying the number of Goblet cells in each villus across the three intestinal segments. (h) Representative immunohistochemically stained sections of the duodenum, jejunum, and ileum from young and old mice, quantifying the number of Enteroendocrine cells in each villus across the three intestinal segments. (i) KEGG pathway enrichment analysis of ISC‐related genes identified in the single‐cell sequencing results of young and old mice. The staining experiments included six samples per group (*n* = 6). Data are presented as mean ± SD, and a Student's *t*‐test was utilized for statistical analysis. Significance levels are indicated as follows: *p < 0.05, **p < 0.01, and ***p < 0.001. The age of the young mice was 2–3 months, whereas the aged mice were 28 months old.

Previous results have demonstrated that the functionality of the small intestine declines with advancing age, accompanied by alterations in the epithelial structure. Consequently, our team undertook single‐cell sequencing of senescent intestinal epithelial cells to explore these changes in greater depth. The UMAP plot illustrates that aged mice exhibit a decrease in intestinal absorptive cells, a reduction in goblet cells (Figure 2a), and a decline in the population of intestinal stem cells and progenitor cells within the epithelial compartment (Figure 2b). These observations were validated through immunohistochemical staining (Figure 2c,d). Subsequently, we evaluated the functions of ISC, concentrating on regeneration, proliferation, and differentiation. Initially, we noted a significant decline in the capacity of crypts to form organoids with aging, characterized by reduced budding and the formation of large sac‐like structures (Figure 2e). Simultaneously, the proliferative ability of the crypts was significantly diminished (Figure 2f). Additionally, the proportions of various differentiated cell types exhibited abnormal alterations, including a notable decrease in the number of goblet cells across multiple regions of the small intestine (Figure 2g) and an increase in the number of enteroendocrine cells in the duodenum (Figure 2h). This raises the question: what specific changes have transpired in the aging ISC that have contributed to the modifications in the small intestinal epithelium? To address this inquiry, we further analyzed the single‐cell sequencing data and conducted GSEA on the mutant genes within the ISC population. The findings indicated that the epithelial changes associated with senescence were closely associated with the metabolic pathways of ISC (Figure 2i).

### 3.3 The Metabolic Activity of ISC Diminishes in Aged Mice

**FIGURE 3:**
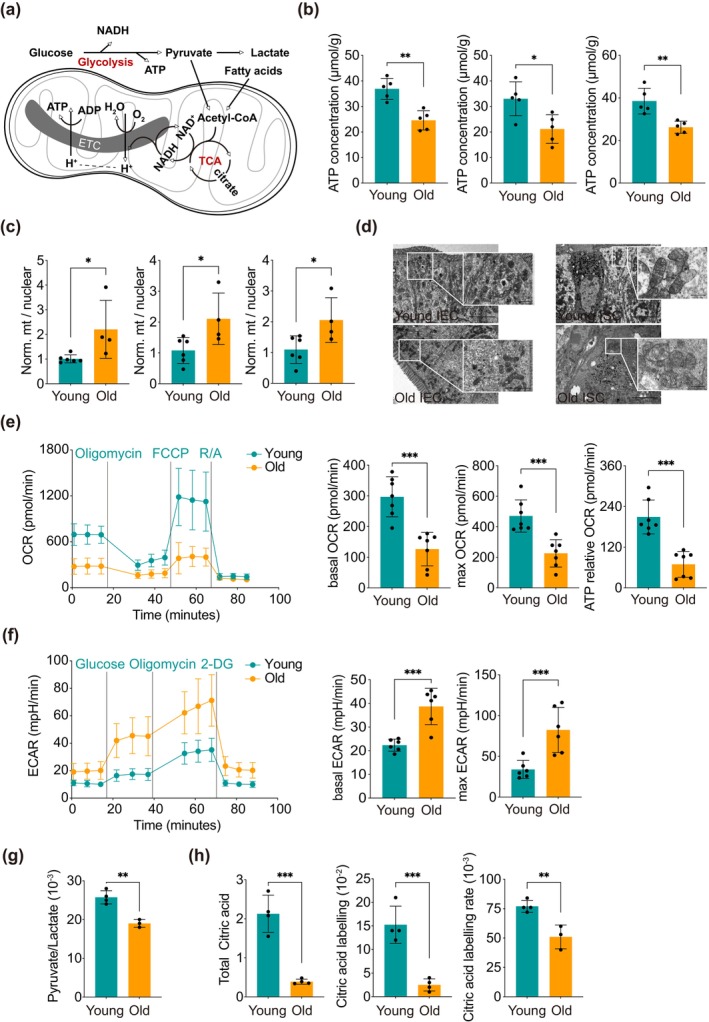
Mitochondrial metabolism in ISCs is disrupted in older mice, resulting in inadequate energy supply. (a) Schematic diagram illustrating mitochondrial metabolism, encompassing OXPHOS, glycolysis, and FAO. (b) ATP production levels in the duodenum, jejunum, and ileum epithelial cells of young and old mice. (c) Quantification of mitochondrial DNA copy number in ISCs from young and old mice, with *n* = 4–6 mice. (d) Transmission electron microscopy images depicting mitochondrial structure and morphology in small intestinal epithelial cells and ISCs from both young and old mice. Scale bars: 5 μm, 1 μm, and 500 nm. (e) OCR measured by a mitochondrial stress test (Seahorse) in ISCs from young and old mice, with organoids derived from at least six mice. Results were validated in at least three independent experiments. (f) Glycolytic capacity of ISCs from young and old mice assessed using a glycolysis stress test (Seahorse), with basal ECAR determined. (g) Ratio of pyruvate to lactate measured in ISCs from young and old mice, with 3–4 mice per group. (h) Citrate content and labeling rate in the crypts of young and old mice, labeled with [U‐^13^C] palmitate, with 3–4 mice per group. Data are presented as the mean ± SD. A Student's *t*‐test was employed for statistical analysis. *p < 0.05, **p < 0.01, ***p < 0.001. The age of the young mice was 2–3 months, while the aged mice were 28 months old.

GSEA pathway enrichment analysis indicated a significant association between alterations in senescent intestinal epithelium and the ISC metabolic pathway, which is reliant on mitochondrial energy metabolism (Figure 3a). To further explore the modifications in mitochondrial energy metabolism within aging ISCs, our initial focus was on energy production. We evaluated the energy‐generating capacity across various segments of the small intestine and found that the senescent intestinal epithelium produced a lower quantity of ATP (Figure 3b) and exhibited an abnormal accumulation of mtDNA copy number in senescent ISC (Figure 3c). Employing electron microscopy, we analyzed the quantity and morphology of mitochondria, revealing that young IECs and ISCs contained a greater number of mitochondria with well‐defined internal cristae. In contrast, aging was associated with a reduced number of mitochondria, which displayed increasingly rounded and spherical morphologies, alongside the disruption and degradation of internal cristae (Figure 3d). The morphology of mitochondria is critically linked to their metabolic preferences, and our findings suggest that aging ISCs exhibit distinct metabolic characteristics. Senescent ISCs exhibited significantly diminished oxygen consumption (Figure 3e) and enhanced glycolytic activity (Figure 3f), as quantified by hippocampal energy analyzer. Furthermore, the pyruvate/lactate ratio in senescent ISCs was found to be lower, as determined by LC–MS analysis (Figure 3h). These results imply that mitochondrial function in ISCs becomes dysregulated with senescence, resulting in a decreased OXPHOS capacity and an increased dependence on glycolysis. It is noteworthy that glycolysis is considerably less efficient in ATP production compared to OXPHOS, leading to a reduced energy production capacity in aging ISCs.

In addition to glycolysis, FAO is a vital pathway for cellular energy metabolism. To further substantiate the decline in FAO (Mihaylova et al. 2018), we conducted a metabolomics study utilizing labeled substrates to evaluate substrate utilization by FAO. Following incubation with [U‐^13^C]palmitate, both the total citrate concentration in the crypt and the citrate labeling rate demonstrated significant reductions (Figure 3i). Based on these findings, we conclude that the mitochondrial function of ISC is compromised in the aging state, resulting in an inadequate energy supply and diminished metabolic capacity.

### 3.4 ADF Modulates the Metabolic Processes of ISC

**FIGURE 4:**
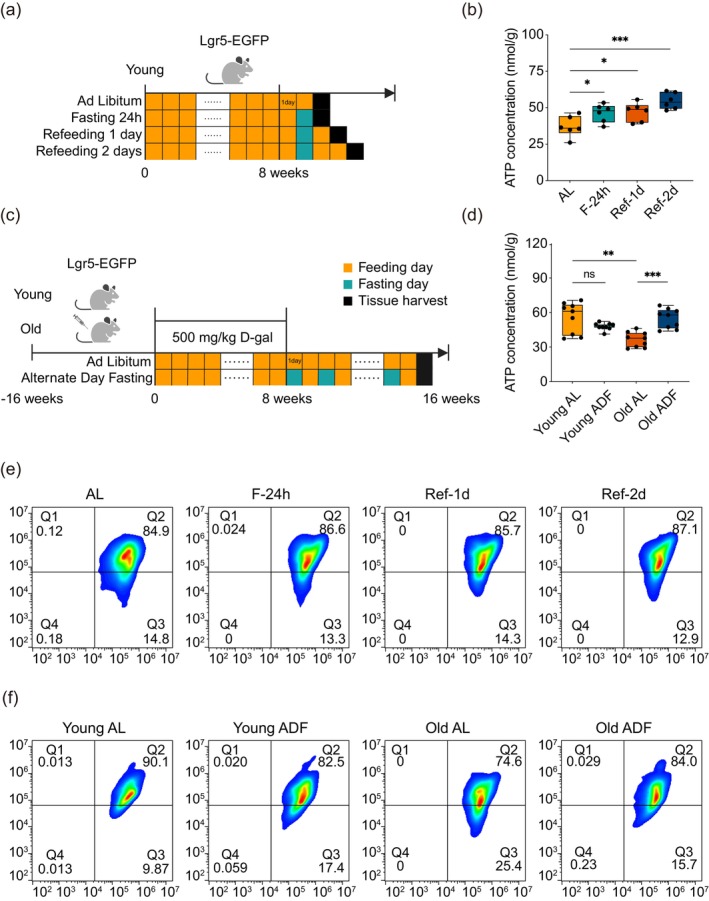
The ADF dietary model can regulate ISC mitochondrial metabolism. (a) Schematic diagram illustrating short‐term ADF treatment in young Lgr5‐EGFP mice. (b) Concentration of ATP production in small intestinal epithelial cells from young Lgr5‐EGFP mice. (c) Schematic diagram depicting long‐term (8 weeks) ADF treatment in both young and aged Lgr5‐EGFP mice. (d) Concentration of ATP production in small intestinal epithelial cells from young and aged Lgr5‐EGFP mice. (e) Flow cytometry results showing the JC‐1 mitochondrial membrane potential of ISCs in young mice. (f) Flow cytometry results illustrating the JC‐1 mitochondrial membrane potential of ISCs in both young and aging model mice. Each group in the experiment consisted of 6–10 mice. The data are presented as the mean ± standard deviation. A Student's *t*‐test was employed for statistical analysis, with significance levels indicated as follows: *p < 0.05, **p < 0.01, ***p < 0.001. Young represents 2–3 month‐old mice, and Old represents aging‐model mice.

In a state of fasting, the capacity of stem cells to metabolize fatty acids for energy sustains their viability during periods of nutrient deprivation. To examine the potential activation of the mitochondrial metabolic program upon refeeding, we classified the subjects based on various dietary patterns (Figure 4a,c) to evaluate ATP production. The data revealed a consistent increase in ATP production in young mice following a brief cycle of ADF (Figure 4b). Notably, in aging model mice subjected to an ADF regimen for up to 8 weeks, ATP production also exhibited an increase, whereas no significant alterations were detected in ATP production among the young mice (Figure 4d). This observation implies that ADF may more effectively enhance the mitochondrial energy‐producing capacity in aged ISCs. Furthermore, we assessed the mitochondrial membrane potential through JC‐1 flow cytometry analysis. The results indicated that short‐term ADF had a negligible effect on the mitochondrial membrane potential of ISCs (Figure 4e). Conversely, the long‐term ADF regimen positively affected the mitochondrial membrane potential of ISCs in aging model mice, resulting in enhanced activation of this potential (Figure 4f). Collectively, these findings suggest that the ADF dietary pattern can modulate the mitochondrial metabolism of intestinal stem cells.

### 3.5 ADF Promotes the Regenerative Potential of ISC in Aged Mice

**FIGURE 5:**
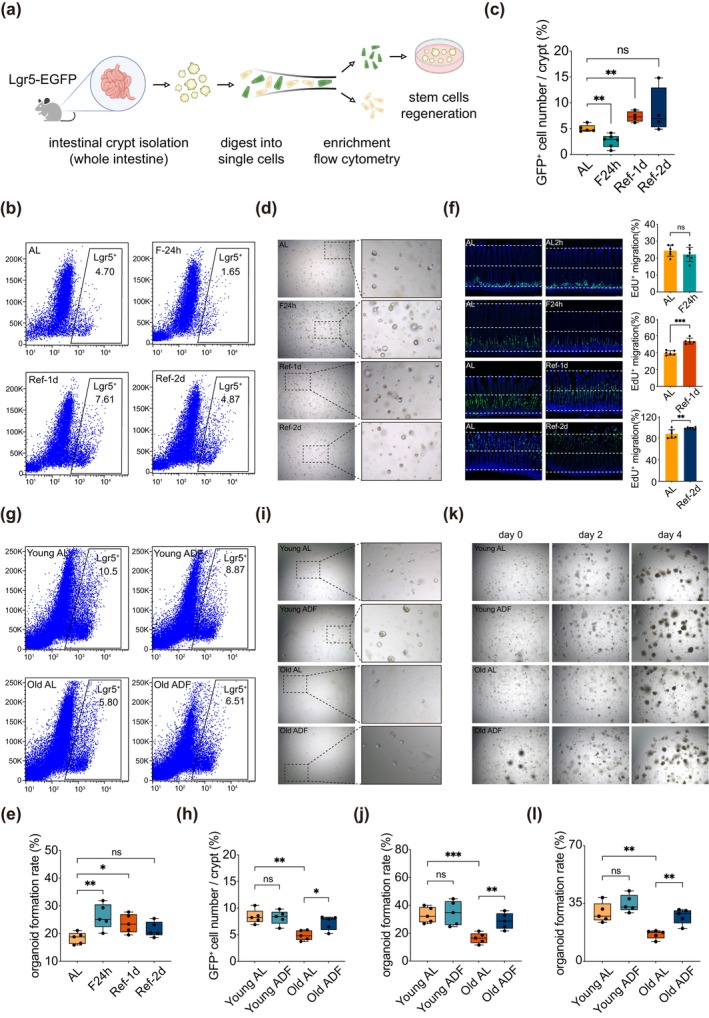
The ADF dietary pattern enhances the regenerative function of aging ISCs. (a) Schematic diagram illustrating the flow sorting process of Lgr5^+^ ISCs from mice. (b, c) Changes in the number of Lgr5^+^ ISCs in young mice following a short‐term ADF dietary intervention, along with statistical results. A total of 5–6 mice were used per group. (d, e) Representative images of ISC organoids and statistical results regarding the regeneration rate of ISCs in young mice after the short‐term ADF dietary intervention. (f) Representative results of EdU^+^ migration staining at 2, 24, 48, and 72 h post‐EdU injection during the short‐term dietary intervention. Six mice were used per group. (g, h) Changes in the number of Lgr5^+^ ISCs in both young and aged mice following a long‐term ADF dietary intervention, along with statistical results. A total of 5–6 mice were used per group. (i, j) Representative images depicting the number of Lgr5^+^ ISCs and statistical results of the long‐term ADF dietary intervention in young and aged mice. (k, l) Representative images and statistical results of organoids formed from the ISC in small intestines of young and old mice. The data are presented as the mean ± standard deviation. A Student's *t*‐test was employed for statistical analysis, with significance levels indicated as follows: *p < 0.05, **p < 0.01, and ***p < 0.001. Young represents 2–3 month‐old mice, and Old represents aging‐model mice.

Intermittent fasting has been demonstrated to improve the functionality of adult stem cells across various tissues. To achieve a more comprehensive understanding of the impacts of both short‐term and long‐term ADF on ISC function, we initially performed flow cytometry sorting on the small intestines of young Lgr5‐EGFP mice. This methodology enabled us to evaluate the capacity of the isolated Lgr5^+^ ISCs to generate self‐renewing organoids (Figure 5a). Notably, the population of ISCs subjected to a 24‐h fasting period was significantly reduced compared to the control group that had unrestricted access to food (Figure 5b). However, the organoid‐forming capability of these ISCs was significantly enhanced (Figure 5d). In the short‐term ADF cohort, the number of ISCs initially increased before stabilizing (Figure 5c), while their ability to form organoids remained significantly elevated (Figure 5d,e). Subsequently, we conducted an EdU migration experiment, which indicated that a 24‐h fasting period had a minimal effect on the rate of intestinal epithelial self‐renewal. Conversely, short‐term ADF significantly accelerated this rate (Figure 5f). These findings suggest that the ADF regimen not only capitalizes on the beneficial effects of fasting but also enhances ISC self‐renewal during the feeding phase, thereby promoting tissue repair. To further elucidate the benefits of long‐term ADF, we assessed its effects on ISC function in both young and aged subjects. Our results revealed that in an aging context, the number of Lgr5^+^ ISCs experienced a significant decline (Figure 5g,h), and their organoid‐forming ability was nearly eliminated (Figure 5i). However, following an extended period of ADF, there was a partial recovery in the number of senescent Lgr5^+^ ISCs (Figure 5g,h), accompanied by a corresponding enhancement in their organoid‐forming capacity (Figure 5i). This recovery was further substantiated by the successful isolation of intestinal crypts capable of forming organoids in vitro (Figure 5j–l).

### 3.6 ADF Enhances the Functional Performance of the Small Intestine in Aged Mice

**FIGURE 6:**
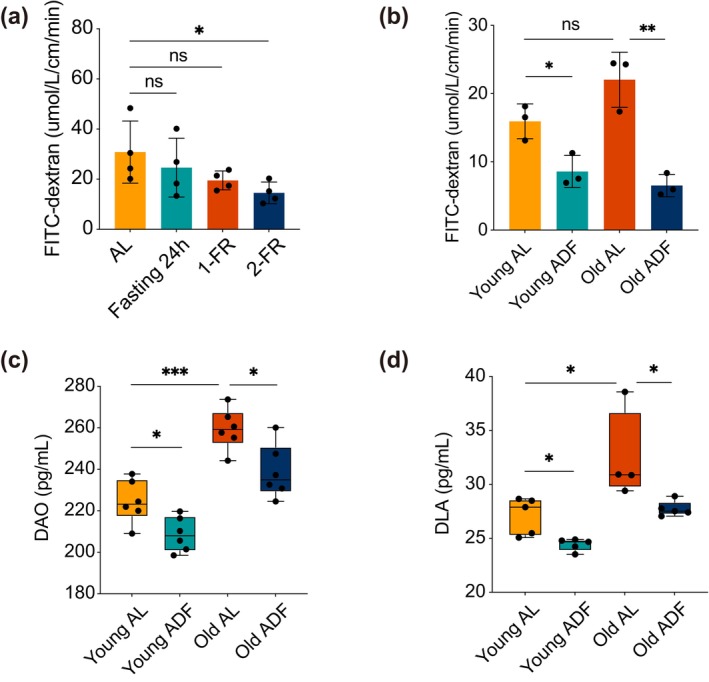
The ADF dietary pattern enhances small intestinal barrier function in aged mice. (a) The ability of young and aged mice to selectively permeate FITC‐Dextran in the small intestine following a short‐term ADF diet intervention. Four mice were used per group. (b) The ability of young and aged mice to selectively permeate FITC‐Dextran in the small intestine after a long‐term ADF diet intervention. Three mice were used per group. (c) The concentration of DAO in the serum of young and aged mice after a long‐term ADF diet intervention. Six mice were used per group. (d) The concentration of DLA in the serum of young and aged mice after a long‐term ADF diet intervention. Four to six mice were used per group. The data are presented as the mean ± standard deviation. A Student's *t*‐test was employed for statistical analysis, with significance levels indicated as follows: *p < 0.05, **p < 0.01, and ***p < 0.001. Young represents 2–3 month‐old mice, and Old represents aging‐model mice.

Prior investigations have suggested that ADF may interfere with the metabolism of ISCs and promote the regeneration of aging ISCs. Therefore, the present study seeks to determine whether the functionality of the aging intestinal epithelial barrier can be improved. To achieve this objective, we evaluated the selective permeability of the small intestine and found that aging mice subjected to D‐galactose modeling exhibited increased permeability. The reduction in permeability observed after a short‐term ADF intervention was not particularly significant (Figure 6a). However, following an extended ADF regimen, a marked enhancement in the permeability of the senescent small intestine was observed (Figure 6b), indicating that the integrity of the intestinal barrier was improved through the stimulation of ISC proliferation. Furthermore, we quantified the levels of DAO and D‐lactate acid (DLA) in serum (Figure 6c,d). DAO, a highly active enzyme located in the cytoplasm of mammalian intestinal villous cells, serves as an indirect marker of intestinal mucosal damage when the integrity of the intestinal mucosa is compromised. DLA, a byproduct of bacterial fermentation, can also reflect alterations in intestinal mucosal permeability, particularly when bacteria that produce DLA translocate into the bloodstream through a compromised barrier. The findings from these two biomarkers confirmed that the intestinal barrier in aging mice was significantly impaired yet exhibited considerable recovery and regeneration following ADF.

## 4 Discussion

Epithelial regeneration is essential for preserving intestinal barrier integrity and optimizing nutrient absorption. A decline in regenerative capacity is a notable marker of intestinal aging. Our research reveals that the intestinal barrier in naturally aging mice is compromised, leading to impaired digestion and nutrient absorption, which indicates a deterioration in the regenerative capacity of the aging intestinal epithelium. This decline in epithelial regeneration is primarily linked to the reduced functionality of ISCs as a result of aging. Our data show a decrease in the number of ISCs throughout the aging process, alongside a significant reduction in the proliferative capacity of intestinal crypts. Corresponding structural changes in the intestine include elongated villi in the duodenum, shortened crypts, and a decrease in goblet cell numbers, while the quantity of enteroendocrine cells in the duodenum increases. These observations are not entirely consistent with previous studies. Although there is ongoing debate regarding the changes in ISC numbers with age (Moorefield et al. 2017; Nalapareddy et al. 2017), it's clear that the proliferation and regenerative potential of ISCs in older mice are significantly diminished (Pentinmikko et al. 2019). Impaired ISC regeneration and dysregulation of the cell cycle lead to substantial alterations in the cellular composition and structure of the intestinal epithelium. Supporting evidence indicates an increase in the size of intestinal crypts, the length of villi, the count of Paneth cells, and the number of goblet cells in naturally aging mice (Gebert et al. 2020). Additionally, it has been documented that the structure of intestinal villi deteriorates with age, characterized by a reduction in both the number and height of crypts (He et al. 2020). Another investigation utilizing a senescence‐accelerated mouse strain, which exhibits a distinct senescence phenotype, assessed age‐related changes in the jejunum and ileum, revealing elongation of both ileal villi and crypt depth, while the height of jejunal villi remained relatively unchanged (Suzuki et al. 2022). We hypothesize that the limited application of mouse models of natural aging, particularly the 28‐month‐old mice used in this study, may be influenced by variables such as gender, age, and strain, which are pertinent considerations in this context.

The main reason for the aging traits seen in the small intestinal epithelium is the reduced ability of ISC to regenerate, which is significantly dependent on energy metabolism, especially mitochondrial function in the ISC. Mitochondrial dysfunction can lead to significant impairments in cellular energy conversion, particularly in tissues that heavily depend on mitochondrial activity for energy, such as the small intestine. During the aging process, the efficacy of OXPHOS and antioxidant defense mechanisms diminishes, resulting in prolonged depletion of the electron transport chain (ETC). This condition promotes the production of reactive oxygen species (ROS) and mutations in mtDNA, ultimately culminating in cell death (Amorim et al. 2022). Our experimental findings support these observations, indicating that the OXPHOS capacity and ATP production of ISCs in aged mice are significantly reduced, with mtDNA accumulation observed in these cells as they age. Furthermore, nutrient sensing may also become dysregulated with aging. The nutrient sensing pathway encompasses IGF‐1, the mechanistic target of mTOR‐S6 pathway, FOXO, and members of the AMPK family. These pathways interact to sustain metabolic homeostasis. AMPK functions as a central nutrient sensor that regulates mTOR signaling, activates the FOXO transcription factor, and influences SIRT1 (Saxton and Sabatini 2017). SIRT1 can, in turn, activate FOXO and PGC1α, both of which are critical for mitochondrial metabolic processes (Brunet et al. 2004; El‐Khamisy et al. 2005). An increase in the glycolysis to OXPHOS ratio may also induce changes in the NAD/NADH ratio, further reducing SIRT activity (Chakravarti et al. 2021). This observation is consistent with our data, which demonstrate a significant enhancement in the glycolytic capacity of ISCs in aged mice. The metabolic state of ISCs directly impacts their fate, thereby influencing the ability of the intestinal epithelium to maintain homeostasis.

Aging can fundamentally be described as a process characterized by a disparity between energy supply and demand. This disparity may be alleviated through various interventions, including pharmacological agents such as metformin, resveratrol, and rapamycin (Gonzalez‐Freire et al. 2020). Recent progress in aging research has introduced interventions that target cellular and mitochondrial physiology as innovative anti‐aging strategies. Although the mechanism by which caloric restriction promotes longevity is not yet fully understood, the beneficial effects of caloric restriction, as demonstrated in studies involving yeast (Robinson et al. 2002) and *Caenorhabditis elegans* (Schulz et al. 2007), are associated with mitochondrial physiology. However, most previous studies on ISC senescence have predominantly focused on classical signaling pathways, such as the Wnt and Notch pathways, which are essential regulators of senescence, proliferation, and differentiation of ISC (Kurokawa et al. 2020; Ludikhuize et al. 2020; Yan et al. 2017). Specifically, WNT3, EGF, and Notch ligands DLL1 and DLL4 are recognized as fundamental regulators of ISCs (Clevers and Bevins 2013; Ootani et al. 2009). Recent evidence suggests that dietary factors can influence ISCs fate by modulating metabolic pathways and responding to variations in nutritional status (Wang et al. 2021). A study demonstrated that young mice undergoing a 24‐h fasting period displayed enhanced fatty acid oxidation in ISCs and improved ISCs functionality. It has been observed that with advancing age, ISCs function declines, which is associated with a decrease in fatty acid oxidation. Therefore, both fasting and the administration of PPAR‐δ agonist drugs have the potential to promote fatty acid oxidation and augment the activity and proliferation of aging ISCs (Mihaylova et al. 2018). Another study examined the changes in the small intestine of young and aging mice after 4 weeks of a 30% dietary restriction, followed by 2 days of ad libitum feeding. The results indicated that a 30% dietary restriction influences the expression levels of ISCs differentiation markers. Additionally, refeeding impacts the activity of Hmgcs2, the rate‐limiting enzyme in ketogenesis, which subsequently regulates the differentiation pathway of ISCs and can partially restore the cellular composition of intestinal crypts in older mice (Gebert et al. 2020). Notably, recent investigations have demonstrated that a short‐term fasting regimen lasting 1day, followed by 1 day of re‐feeding in young mice, significantly enhances the proliferation and regeneration of Lgr5^+^ ISCs in both the small intestine and colon, albeit with an increased incidence of tumors in the context of Apc mutations. This phenomenon is attributed to the robust activation of the mTORC1 signaling pathway in ISCs during re‐feeding, which promotes the expression of OAT, thereby facilitating polyamine metabolism and enhancing ISCs protein synthesis, ultimately improving ISC proliferation (Imada et al. 2024). In light of the aforementioned findings, rapid refeeding cycles must be meticulously evaluated and tested when developing diet‐based regeneration strategies to avoid elevating cancer risk. This consideration is essential, as rapid refeeding has the potential to activate stem cell‐mediated regeneration, which could result in tumorigenic events. In our study, we implemented an ADF regimen in elderly mice and observed promising results; both short‐term and long‐term ADF improved the self‐renewal rate and regenerative capacity of aging ISCs while also stimulating increased ATP production in these cells. We hypothesize that ADF may delay ISC senescence by influencing mitochondrial metabolism, although further investigation is required to ascertain its effects on mtDNA and OXPHOS.

In conclusion, our study evaluated the alterations in senescent intestinal epithelial cells and the functionality of ISCs to mitigate the deterioration of the intestinal epithelium associated with aging. The implementation of dietary interventions, specifically ADF, in aging murine models provides valuable insights for the dietary management of intestinal health. Such dietary strategies hold significant promise for addressing intestinal disorders, particularly those related to aging; however, further research is necessary to substantiate these findings.

## Author Contributions

**Heng Quan:** investigation, formal analysis, writing – original draft. **Yao Lu:** methodology. **Yingying Lin:** resources. **Peng Xue and Wuqi Yang:** data curation. **Yuning Zhang and Yuqi Wang:** funding acquisition. **Weiru Yu and Xiaoya Lin:** data curation. **Yafei Zhang and Cong Lv:** software. **Fazheng Ren:** writing – review and editing. **Huiyuan Guo:** project administration and supervision. All authors agree to be accountable for the content of the work and approved the manuscript.

## Conflicts of Interest

The authors declare no conflicts of interest.

## Supporting information


Figure S1.
Figure S2.

## Data Availability

The data that support the findings of this study are available from the corresponding author upon reasonable request.
