# Supplementary material for: Alternate Day Fasting Enhances Intestinal Epithelial Function During Aging by Regulating Mitochondrial Metabolism
Source: Aging Cell. 2025 Apr 1;24(7):e70052. doi: 10.1111/acel.70052 (PMC12266747; doi:10.1111/acel.70052)
Supplement: Supplementary file 1 — Figure S1. Figure S2. [file ACEL-24-e70052-s001.pdf]

Supplementary Fig. 1

(a)

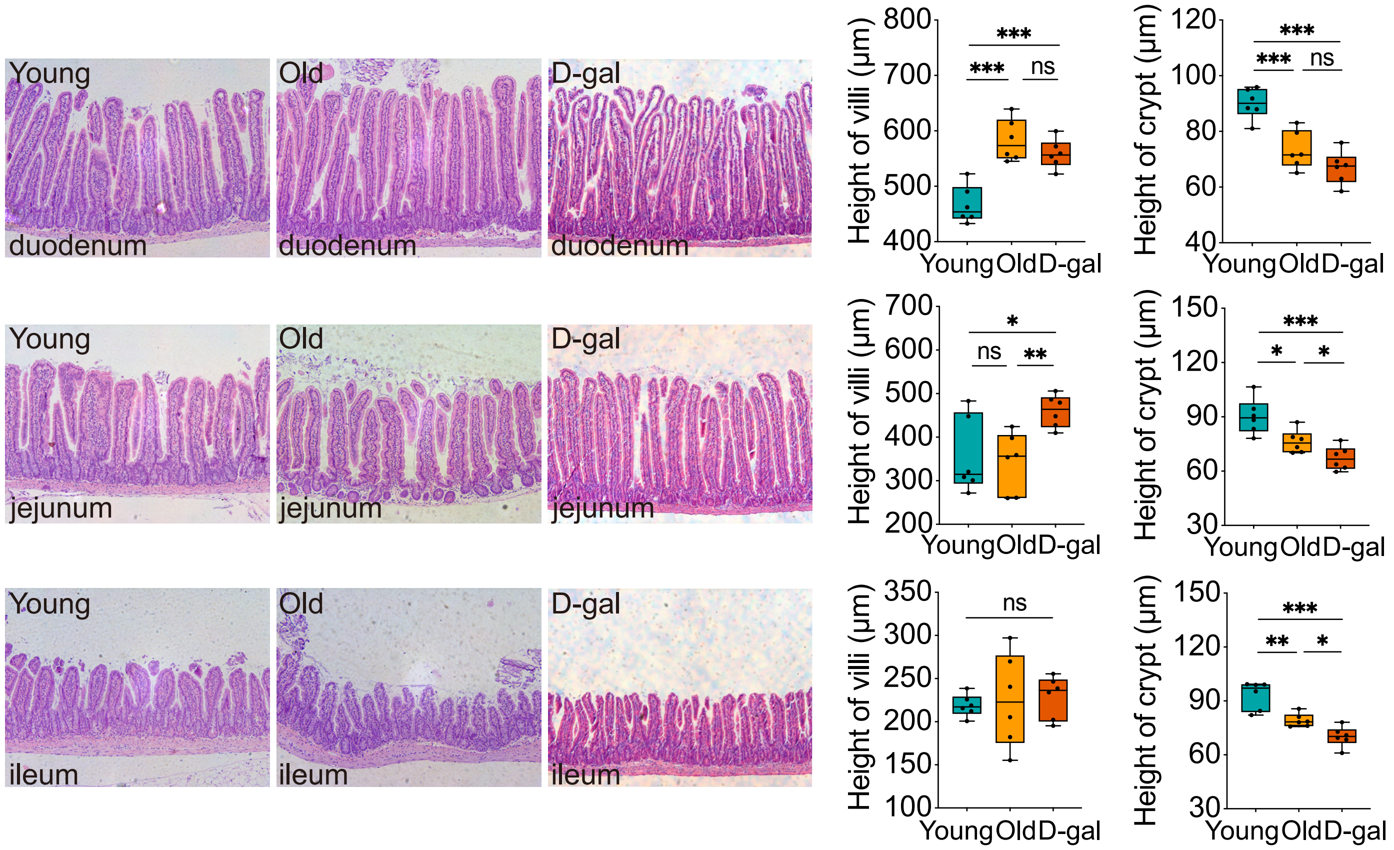

(b)

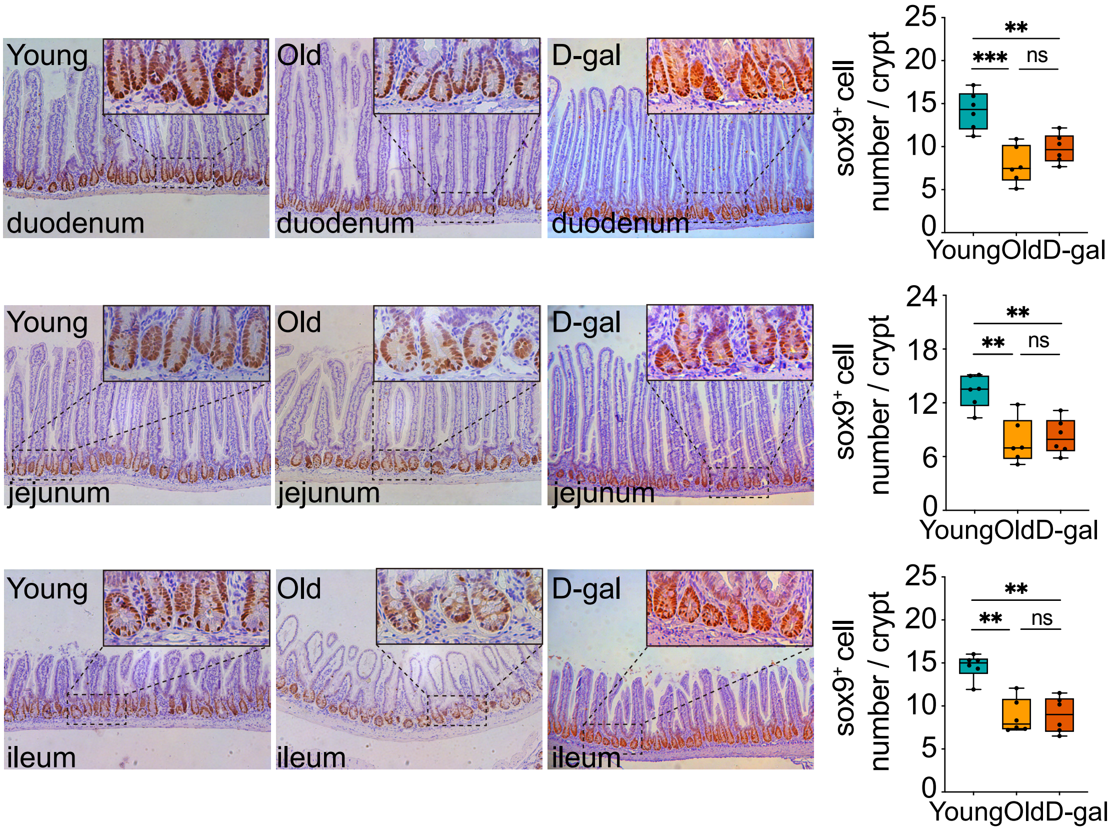

(c)

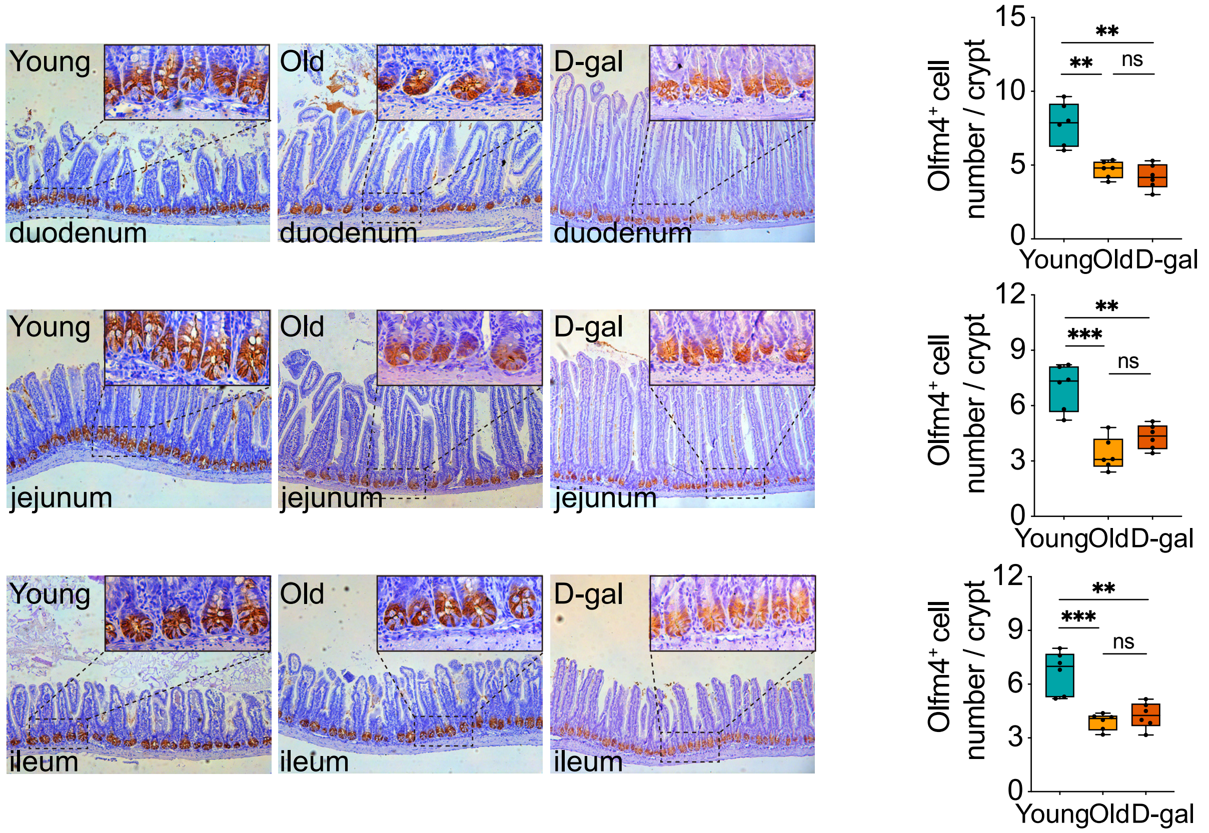
Young
Old
D-gal
duodenum
jejunum
ileum
Olfm4+ cell number / crypt
**
ns
***
Young Old D-gal

(d)

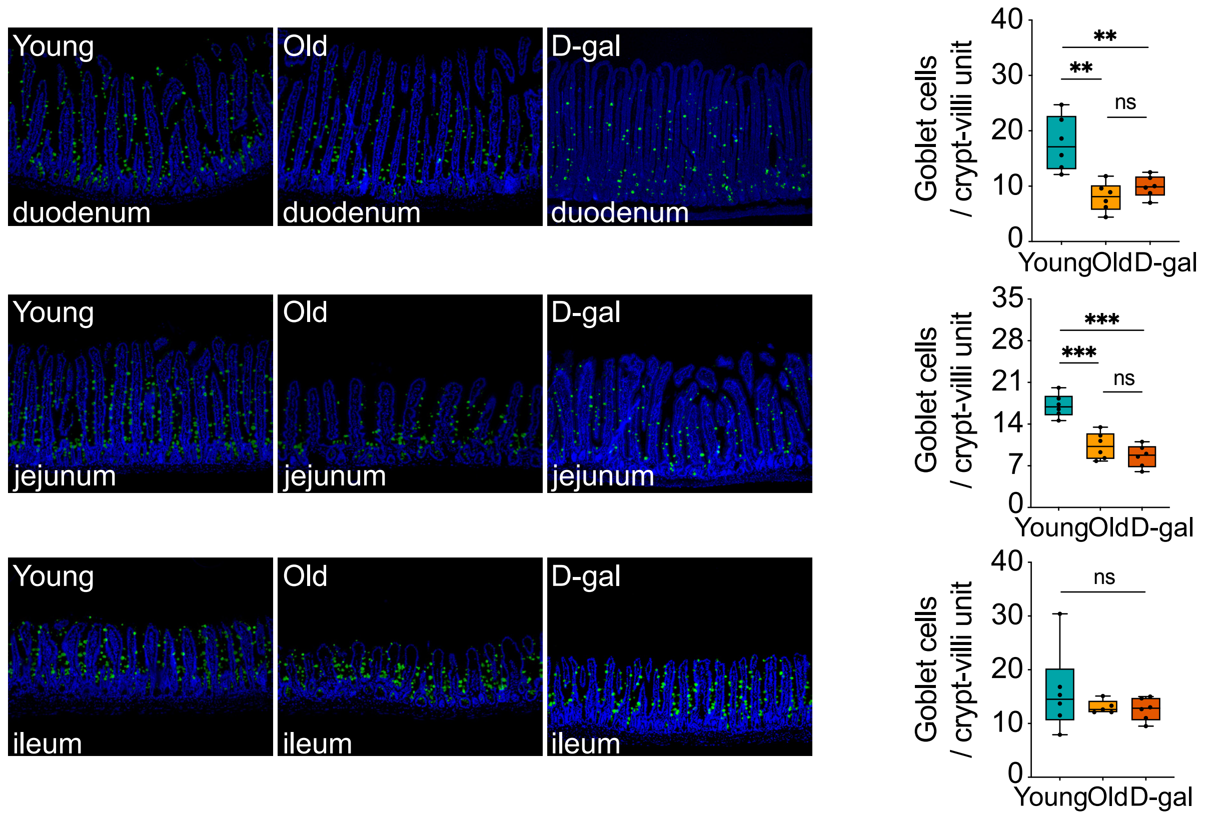
Young
Old
D-gal
duodenum
jejunum
ileum
Goblet cells / crypt-villi unit
**
***
ns
Young Old D-gal

(e)

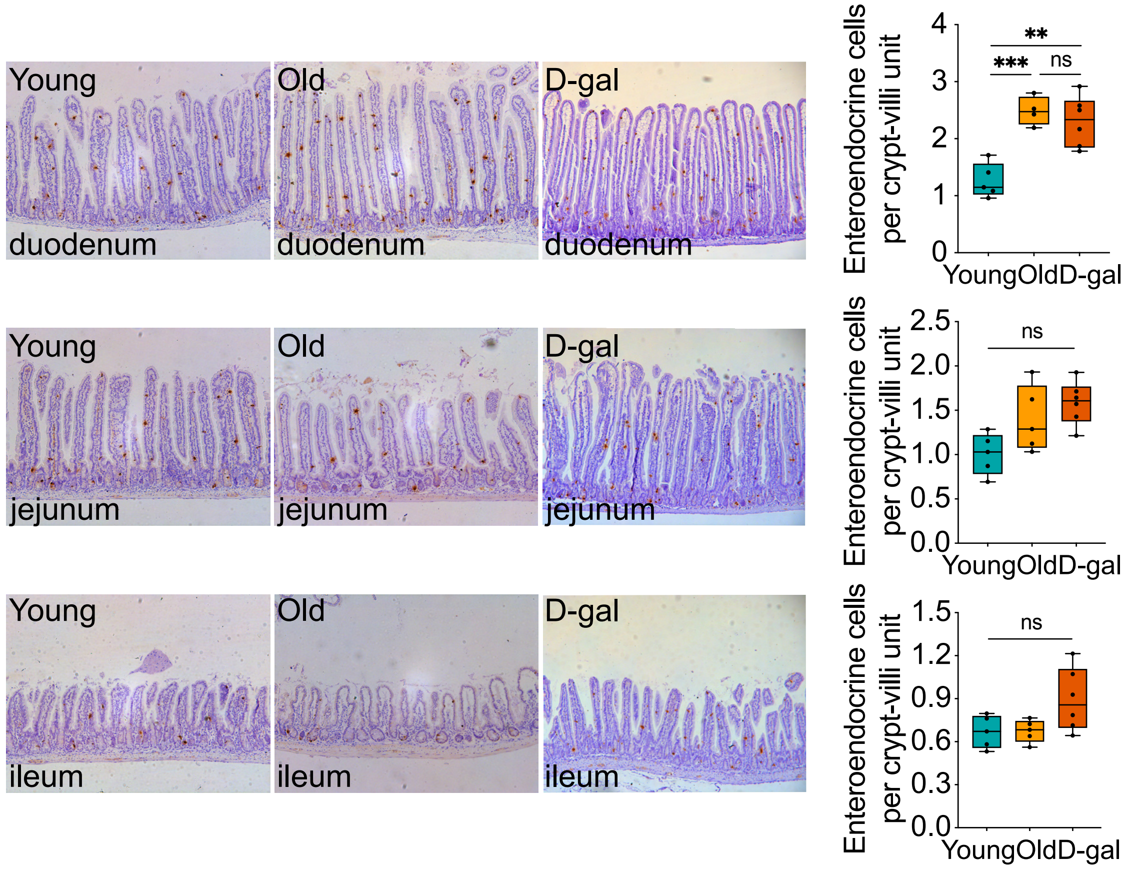
Young
Old
D-gal
duodenum
duodenum
duodenum
Young
Old
D-gal
jejunum
jejunum
jejunum
Young
Old
D-gal
ileum
ileum
ileum
Enteroendocrine cells per crypt-villi unit
4
3
2
1
0
**
***
ns
Young Old D-gal
Enteroendocrine cells per crypt-villi unit
2.5
2.0
1.5
1.0
0.5
0.0
ns
Young Old D-gal
Enteroendocrine cells per crypt-villi unit
1.5
1.2
0.9
0.6
0.3
0.0
ns
Young Old D-gal

(f)

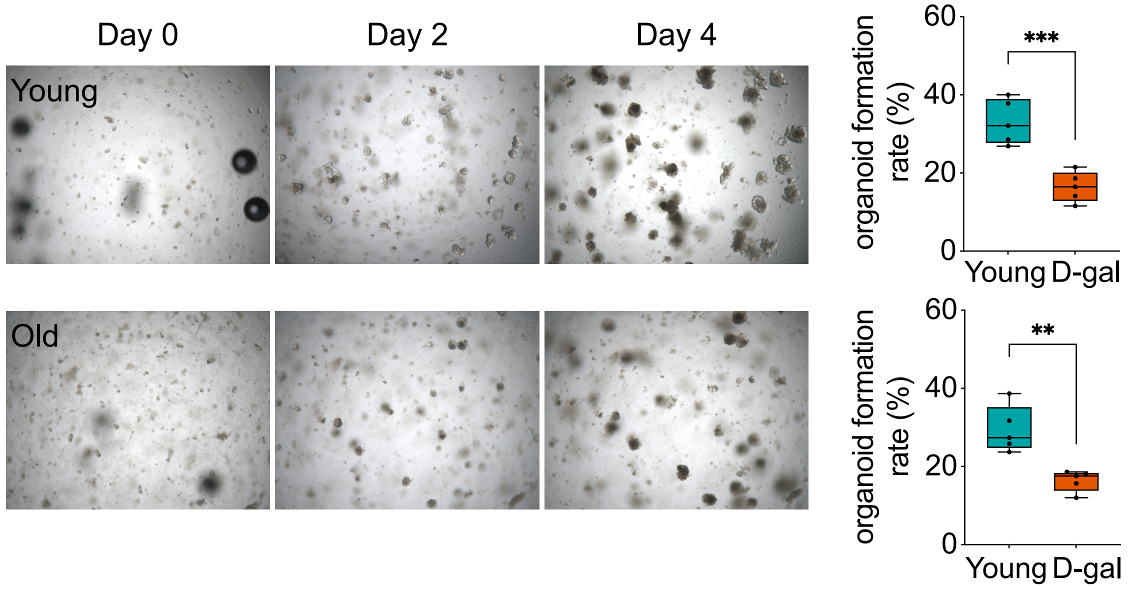
Day 0
Day 2
Day 4
Young
Old
organoid formation rate (%)
60
40
20
0
***
Young D-gal
organoid formation rate (%)
60
40
20
0
**
Young D-gal

Supplementary Fig. 2

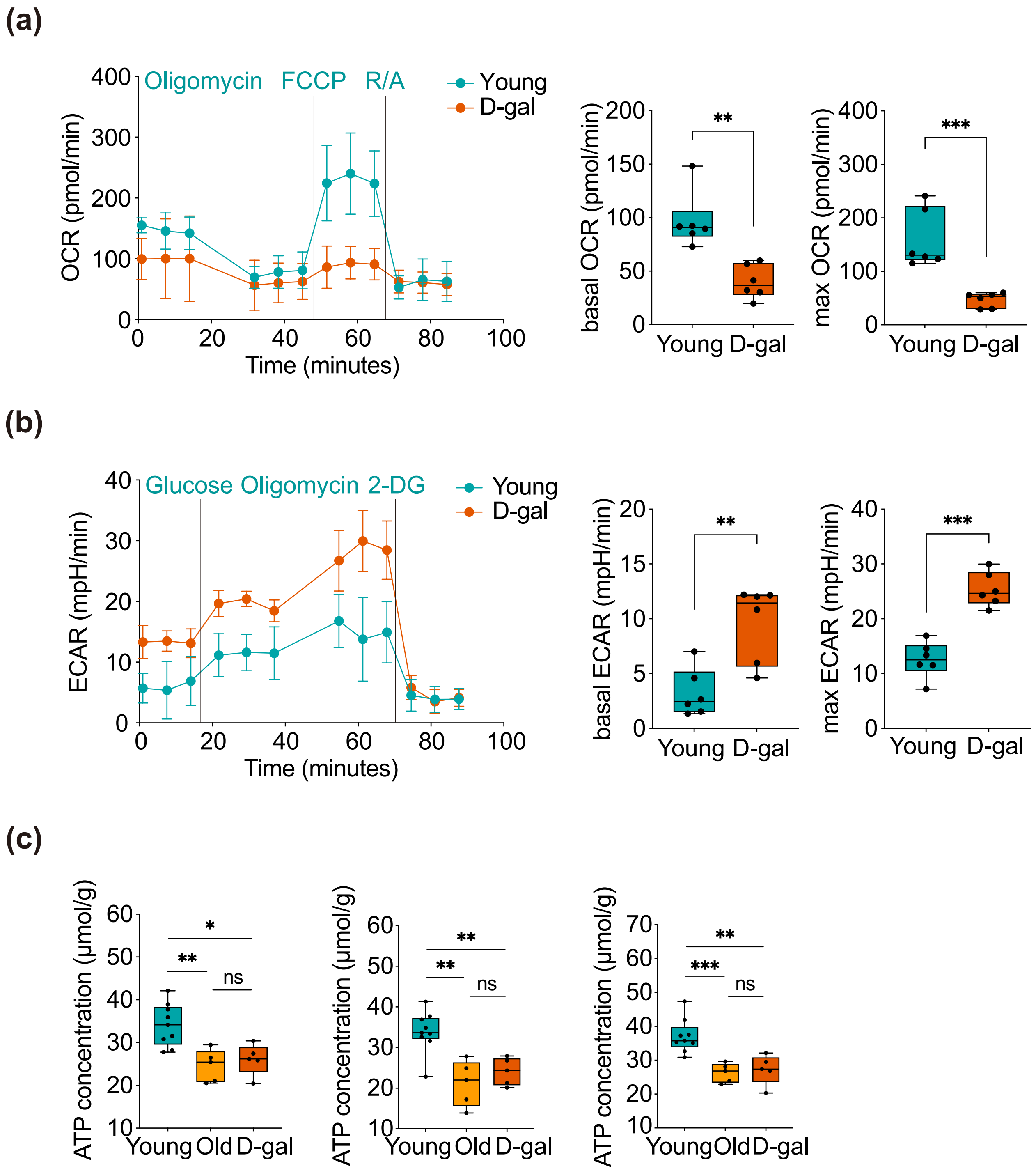

Supplementary Figure captions

Supplementary Figure 1. Degeneration of intestinal epithelial structure in aged and D-gal-induced aging mice. (a) Representative H&E staining of duodenal, jejunal, and ileal sections from Young, Old, and D-gal-induced aging mice. Villus height and crypt depth were quantified across all three intestinal segments. (b) Representative IHC staining of duodenal, jejunal, and ileal sections. $Sox9^{+}$ cells per crypt were quantified. (c) Representative IHC staining of duodenal, jejunal, and ileal sections. $Olfm4^{+}$ cells per crypt were quantified. (d) Representative IF staining of duodenal, jejunal, and ileal sections. Goblet cells per villus were quantified. (e) Representative IHC staining of duodenal, jejunal, and ileal sections. Enteroendocrine cells per villus were quantified. (f) Representative images and regeneration rates of intestinal organoids derived from ISCs of Young, Old, and D-gal-induced aging mice. For all experiments described above, each group consisted of n = 6. Data are presented as mean ± SD. Statistical significance was assessed using Student's t-test, with significance levels denoted as follows: *$P < 0.05$, **$P < 0.01$, and ***$P < 0.001$. Experimental groups were defined as: Young: 2-3-month-old mice, Old: 28-month-old naturally aged mice, D-gal: D-galactose-induced aging model mice.

Supplementary Figure 2. Mitochondrial metabolic dysfunction and energy deficiency in ISCs of aging model mice. (a) Oxygen consumption rate (OCR) measured via mitochondrial stress test (Seahorse XFe96) in ISCs isolated from Young and D-gal-induced aging mice. Organoids derived from ⩾6 mice per group (n = 6). Results were validated in ⩾3 independent experiments. (b) Glycolytic capacity assessed by glycolysis stress test (Seahorse XFe96), with basal extracellular acidification rate (ECAR) quantified. (c) ATP concentration in epithelial cells of duodenum, jejunum, and ileum from Young and D-gal-induced aging mice. The data are presented as mean ±SD. Statistical significance was determined using Student's t-test, with significance levels denoted as *$P < 0.05$, **$P < 0.01$, and ***$P < 0.001$. Experimental groups were defined as follows: Young: 2-3-month-old mice, Old: 28-month-old naturally aged mice, D-gal: D-galactose-induced aging model mice.
